# Glutathione‐Related Metabolite Levels and Enzyme Activities in Depression: A Systematic Review and Meta‐Analysis

**DOI:** 10.1002/npr2.70125

**Published:** 2026-04-30

**Authors:** Takahide Etani, Nobuaki Hondo, Sho Moriguchi, Kazunari Yoshida, Sotaro Moriyama, Koki Takahashi, Tahichi Moritake, Yumina Nakamura, Ryoma Kani, Shiori Honda, Masataka Wada, Sakiko Tsugawa, Yukino Mori, Hikari Segawa, Tony Kaku, Hiroyuki Uchida, Shinichiro Nakajima

**Affiliations:** ^1^ Keio University Hospital Shinjuku‐ku Tokyo Japan; ^2^ Japanese Red Cross Ashikaga Hospital Ashikaga‐shi Tochigi Japan; ^3^ Graduate School of Media and Governance Keio University Fujisawa‐shi Kanagawa Japan; ^4^ Department of Neuropsychiatry Keio University School of Medicine Shinjuku‐ku Tokyo Japan; ^5^ National Institutes for Quantum Science and Technology Chiba‐shi Chiba Japan; ^6^ Postgraduate Education Center Kameda Medical Center Kamogawa‐shi Chiba Japan; ^7^ Department of Psychiatry and Behavioral Health, Renaissance School of Medicine Stony Brook University Stony Brook New York USA; ^8^ Department of Psychiatry and Behavioral Sciences Stanford University Stanford California USA; ^9^ Department of Physiology, Graduate School of Medicine Yokohama City University Yokohama‐shi Yokohama Japan; ^10^ National Kohnodai Hospital Medical Center Ichikawa‐shi Chiba Japan; ^11^ National Hospital Organizations Saitama Hospital Wako‐shi Saitama Japan; ^12^ Multimodal Imaging Group, Research Imaging Centre, Centre for Addiction and Mental Health Toronto Ontario Canada

**Keywords:** depression, glutathione, oxidative stress, systematic review and meta‐analysis

## Abstract

Recently, an increasing number of studies have reported abnormal levels of glutathione (GSH)‐related metabolites and enzyme activities in neuroimaging and peripheral samples in patients with depression, indicating abnormalities in the GSH cycle in patients with depression. However, the abnormalities in the GSH cycle have not been comprehensively investigated. Here, we conducted a systematic review and meta‐analysis to compare the levels of GSH‐related metabolites (GSH and GSH disulfide [GSSG]) and the activities of GSH‐related enzymes (Glutathione‐peroxidase [GPx], GSH reductase [GR], glutamate‐cysteine ligase [GCL], GSH synthetase [GS], and GSH S‐transferase [GST]) between patients with depression and healthy controls (HC). The search was performed with MEDLINE, Embase, and PsychINFO. The inclusion criteria were studies comparing levels of GSH‐related metabolites and enzyme activities between patients with depression and HC. Standardized mean differences were calculated to assess group differences. Thirty studies met the eligibility criteria, which included 1019 patients and 947 HC. Our meta‐analysis indicated significantly decreased activity of GPx in patients with depression compared with HC, while the levels of GSH and GR were not significantly different between the groups. Because no studies, or an insufficient number of studies, have been conducted on GSSG, GST, GCL, and GS, meta‐analyses were not performed for these metabolites and enzyme activities. Our findings suggest that abnormal cycling of the GSH system, particularly decreased GPx activity, is involved in the pathophysiology of depression and may serve as a potential biomarker for this illness. Furthermore, given that no or insufficient studies have been conducted on GSSG, GST, GCL, and GS, this study highlights the need for further research to fully elucidate the GSH cycle in patients with depression.

## Abstract 2

We conducted a systematic review and meta‐analysis to compare the levels of glutathione‐related metabolites. Our meta‐analysis indicated significantly decreased activity of glutathione‐peroxidase in patients with depression compared with healthy controls, while the levels of glutathione and glutathione reductase were not significantly different between the groups.

## Graphical abstract

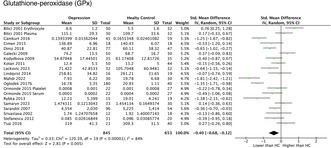

## 1 Introduction

Major depressive disorder (MDD) is one of the most devastating mental disorders worldwide [1, 2, 3], with reduced quality of life [4] and a high recurrence rate [5, 6]. Although several antidepressants [7, 8, 9] and psychotherapy [10, 11, 12] are recognized as effective treatments, they are only sufficiently effective in a limited number of patients. Therefore, it is a prerequisite to understand the pathophysiology of depression to establish its biomarkers and identify its viable therapeutic targets.

**FIGURE 1:**
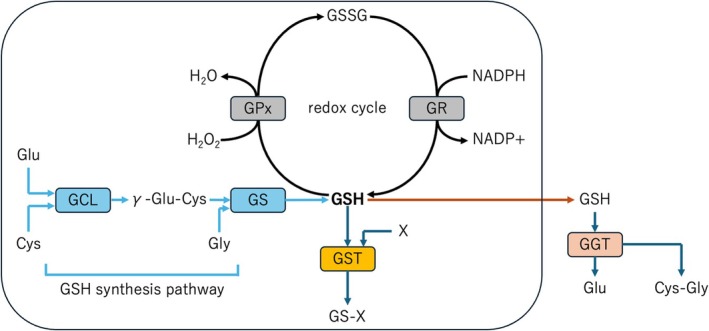
The synthesis pathway of GSH. The synthesis pathway of GSH is provided based on Tsugawa et al. [21]. This pathway involves two enzymatic steps catalyzed by GCL and GS, both of which require coupled ATP hydrolysis. First, GCL combines glutamate (Glu) and cysteine (Cys) to form γ‐glutamylcysteine (γ‐Glu‐Cys). Subsequently, GS catalyzes the addition of glycine (Gly) to this dipeptide, producing GSH. GCL activity is considered the rate‐limiting step in this pathway because it is regulated by low intracellular cysteine levels and negative feedback from GSH. The protective function of GSH is exemplified in its role in reducing hydrogen peroxide and organic peroxides. GSH acts as a cofactor for enzymes such as GPx and GST, facilitating the conversion of toxic oxides into oxidized dimeric GSSG. GSSG is subsequently reduced back to GSH by GR, using NADPH as an electron donor. Under conditions of high oxidative stress, the oxidation of GSH increases, leading to the accumulation of GSSG. To maintain cellular oxidative balance, GSSG is actively transported out of the cell. At this stage, GGT converts extracellular GSH into γ‐glutamylamino acids and cysteinylglycine (Cys‐Gly). These degraded peptides are then transported back into the cell, supporting the recycling and maintenance of intracellular GSH levels. ATP, adenosine triphosphate; GCL, glutamate‐cysteine ligase; GGT, ecto‐enzyme γ‐glutamyltranspeptidase; GPx, glutathione peroxidase; GR, glutathione reductase; GS, GSH synthetase; GSH, glutathione; GSSG, GSH disulfide; GST, GSH S‐transferase; NADPH, nicotinamide adenine dinucleotide phosphate.

Recently, there has been mounting evidence reporting an abnormal glutathione (GSH) cycle in patients with depression [13]. GSH is a major antioxidant that plays multiple roles in maintaining redox balance and protecting cells from oxidative stress and toxicity of xenobiotic electrophiles [14]. The synthesis pathway consists of two steps by glutamate‐cysteine ligase (GCL) and GSH synthetase (GS), which requires coupled adenosine triphosphate (ATP) hydrolysis (Figure 1). Firstly, GCL combines glutamate (Glu) and cysteine (Cys) to form γ‐glutamylcysteine (γ‐Glu‐Cys), and then GS catalyzes the binding of this dipeptide to glycine (Gly) to produce GSH. GCL activity is considered a rate‐limiting step because it is regulated by low intracellular cysteine levels and negative feedback by GSH. The protective function of GSH is demonstrated in the reduction cycle of hydrogen peroxide and organic peroxides. GSH assists in the reduction of toxic oxides as a cofactor for GSH peroxidase (GPx) or GSH S‐transferase (GST), leading to the oxidized dimeric GSH disulfide (GSSG) [15]. GSSG is then reduced to GSH by GSH reductase (GR) using nicotinamide adenine dinucleotide phosphate (NADPH) as an electron donor. Under conditions of high oxidative stress, GSH oxidation is enhanced and GSSG accumulates. Therefore, GSSG is actively transported out of the cell to maintain the oxidative balance in the cell. At this time, the ecto‐enzyme, γ‐glutamyltranspeptidase (GGT), transfers GSH released from the cell to γ‐glutamyl amino acid and cysteinyl‐glycine (Cys‐Gly). The degraded peptide is then transported back into the cell to maintain the GSH balance.

To our knowledge, four meta‐analyses have examined the role of GSH‐related enzymes in depression. One investigated GSH levels using magnetic resonance spectroscopy (MRS) samples [16], while three focused on GPx activity [13, 17, 18], with one of these also examining GR activity [13]. Regarding GSH levels, a meta‐analysis reported that GSH levels in the occipital cortex were lower in patients with depression compared to healthy controls (HC), whereas no significant differences were found in the medial frontal cortex or when GSH levels across all brain regions were combined [16]. As for GPx activity, Liu et al. [13] explored its levels on erythrocytes (across five studies) and serum (across seven studies) and found no significant difference between patients with depression and HC. However, when two studies were excluded following a sensitivity analysis, an elevation in GPx activity was observed in patients with depression compared to HC. Contrarily, another meta‐analysis on an aggregate sample set (encompassing six studies: four on erythrocytes, one on serum, and one each on whole blood and plasma) found no significant difference in the level of GPx between the two groups [17]. In contrast, the most recent meta‐analysis focusing on unipolar depression (across 12 studies) reported a decrease in GPx activity among patients; nevertheless, this statistical significance disappeared upon the exclusion of one study on adolescents from the analysis [18]. These disparities imply inconsistent conclusions about GPx activity in patients with depression across the previous meta‐analyses. Regarding GR activity, Liu et al. [13] did not observe a significant difference in erythrocyte GR activity between the two groups.

While several meta‐analyses have been undertaken, they bear several limitations. First, although a meta‐analysis on GSH levels in patients with depression reported reduced GSH levels in the occipital cortex of patients compared to HC [16], the analysis was limited to studies using MRS. While this finding is important in highlighting regional reduction in brain GSH levels, it remains unclear whether GSH levels are altered in other sample types, including peripheral samples such as whole blood. Second, conclusions regarding GPx activity have been inconsistent, and the number of studies included in these meta‐analyses remains limited: in relation to GPx activity, prior meta‐analyses included five for erythrocyte [13], seven for serum [13], six [17], and 12 studies [18]. Third, concerning GR activity, Liu et al. incorporated three studies in their meta‐analysis [13]. This issue could be addressed by incorporating additional sample types, including platelet and polymorphonuclear leukocyte samples, and updating the search to include more studies in the meta‐analysis. Fourth, no meta‐analysis has yet been performed on the levels of other GSH‐related metabolite levels and enzyme activities such as GSSG, GST, GCL, and GS, which are also involved in the GSH cycle. Finally, these analyses have not taken into account the potential effects of clinico‐demographic factors such as medical condition, age, sex, and illness severity on metabolite levels and enzyme activities, with the exception of one study [17].

Therefore, to provide a more comprehensive understanding of how the GSH cycle may be altered in patients with depression, and to address the gaps left by previous studies, we conducted a meta‐analysis examining GSH‐related metabolite levels and enzyme activities in patients with depression and related disorders, in comparison to HC. Specifically, we hypothesized that the levels of GSH‐related metabolites and enzyme activities would be abnormal in patients with depression compared with HC. Moreover, we explored the influence of patient characteristics such as medication status, duration of illness, and severity of illness through meta‐regression and subgroup analyses, which were not performed in previous meta‐analyses.

## 2 Patients and Methods

### 2.1 Protocol Registration

The full protocol was uploaded to the International Prospective Register of Systematic Reviews website (CRD42023430900). We have followed the Preferred Reporting Items for Systematic Reviews and Meta‐Analysis (PRISMA) statement [19].

### 2.2 Literature Search

The search was performed with Embase, Medline, and PsycINFO databases. The initial search was carried out by three authors (T.E., N.H., and S.N.) (last search: March 29, 2024) and seven authors (T.E., N.H., S.M., K.Y., S.M., K.T., and S.N.) independently assessed the eligibility of the studies. Twelve authors (T.E., N.H., S.M., K.Y., S.M., K.T., T.M., Y.N., R.K., Y.M., H.S., and S.N.) were involved in the data extraction process, with each author independently verifying the extracted data. Human studies written in English were screened using Embase, Medline, and PsycINFO databases. Discrepancies in study selection were resolved by the authors through discussion.

The literature search for each metabolite or enzyme was conducted using the following terms: “glutathione” or “GSH” for glutathione (GSH) and GSH disulfide (GSSG); “glutamate‐cysteine and ligase” or “gamma‐glutamylcysteine and synthetase” for glutamate‐cysteine ligase (GCL); “glutathione and synthetase” for glutathione synthetase (GS); “glutathione peroxidase” for glutathione peroxidase (GPx); “glutathione reductase” for glutathione reductase (GR); and “glutathione and S‐transferase” for glutathione S‐transferase (GST). These search terms were used in combination with “depressi*” or “mood disorder” to identify relevant articles.

### 2.3 Inclusion Criteria

Full‐length articles written in English were included, which met the following criteria: human studies compared (a) levels of GSH‐related metabolites or enzyme activities between (b) patients with depression (excluding bipolar affective disorder) and (c) HC and (d) the provided dataset enabled calculation of standardized mean differences (SMDs).

### 2.4 Exclusion Criteria

The following articles were excluded: (1) studies without sufficient diagnostic accuracy, (2) genetic studies, (3) studies not about GSH‐related metabolites or enzymes (e.g., GSH‐Hb), (4) postmortem studies, or (5) studies on samples of skin or urine.

### 2.5 Quality Assessment

The quality of the original studies was assessed using the Newcastle‐Ottawa Quality Assessment Scale after arranging it for a cross‐sectional study design [20]. This scale assigns four and two points for patient selection and comparability, respectively. Six points indicated the highest quality, whereas zero points indicated the lowest quality.

### 2.6 Outcome Measures

The primary outcome measures were GSH and GSSG levels following a previous meta‐analysis [21]. The secondary outcome measures were activities of GSH metabolism enzymes (GCL, GS, GPx, GR, and GST).

### 2.7 Data Extraction

The variables recorded from each included study were metabolite levels, enzyme activities, diagnoses, age, sex, antidepressant (medication) status, duration of illness, the score of Hamilton Depression Scale (HAMD‐17) and methods of measurement. If the included studies did not report mean or standard deviation (SD) values of the outcome measures, we asked the authors for additional data. When SD values were not reported and other statistical values were available, these values were transformed to SD according to the Cochrane Handbook for Systematic Reviews of Interventions [22] (http://www.handbook.cochrane.org).

### 2.8 Statistical Analysis

#### 2.8.1 Meta‐Analyses

The following analyses were performed using RevMan 5.4 software (the Cochrane Collaboration, 2014, Nordic Cochrane Center, Copenhagen, Denmark). SMDs were used to calculate differences in metabolite levels or enzyme activities between patients and HC. We analyzed the SMDs of GSH and GSSG levels, the activities of GSH metabolism enzymes (i.e., GCL, GS, GPx, GR, and GST) from the samples of MRS, whole blood, plasma, serum, erythrocyte, polymorphonuclear leukocyte, and cerebrospinal fluid (CSF). Effect sizes were classified as small (SMD = 0.2), moderate (SMD = 0.5), and large (SMD = 0.8), with positive values indicating elevated metabolite levels or enzyme activities in the patient group. We calculated effect sizes and two‐sided 95% confidence intervals (CIs) employing the inverse variance statistical method and random effects model to adjust for the study heterogeneity such as patient population or sample sources [23]. The primary analyses regarding levels of GSH metabolites examined the whole samples and investigated sample sources (i.e., central or peripheral) of the metabolites separately. The central group consisted of studies examining ^1^H‐MRS and CSF, while the peripheral group consisted of studies examining whole blood, erythrocyte, plasma, serum, and polymorphonuclear leukocyte. Moreover, the I^2^ statistic was applied to assess study heterogeneity as a measure of the proportion of variance in the summary effect size attributable to heterogeneity, interpreting *I* ^2^ ≥ 50% to indicate significant heterogeneity not attributable to random error [24]. Sensitivity analyses were conducted using the leave‐one‐out method. Potential publication bias was assessed using Egger's regression test for asymmetry of the funnel plots: if identified, the trim‐and‐fill procedure was used [25, 26]. Additional meta‐analyses were conducted to investigate differences in secondary outcomes within the whole, central, and peripheral samples in the same manner as the primary analyses. Meta‐analyses were performed when at least three studies were available.

#### 2.8.2 Moderator Analyses

Moderator analyses were conducted to investigate the influence of study and patient characteristics on the primary and secondary outcomes. Subgroup analyses were performed for materials (MRS, CSF, whole blood, erythrocyte, plasma, serum, and polymorphonuclear leukocyte), medication status (for medicated or unmedicated groups). Subgroup analyses were performed when at least three studies were available. Meta‐regression analyses were conducted for age, proportion of males, proportion of medicated patients, duration of illness, and the score of HAMD‐17. Meta‐regression analyses were performed when at least five studies were available.

The significance levels were set at 0.05/the number of analyses to control the risk of type I and type II errors. The significance levels for the primary and secondary analyses were set at 0.05/5 analyses (=0.01). The significance levels for subgroup analyses and meta‐regression analyses were set at 0.05/8 analyses (=0.00625) and 0.05/5 analyses (=0.01), respectively.

## 3 Results

### 3.1 Characteristics of Included Studies

**FIGURE 2:**
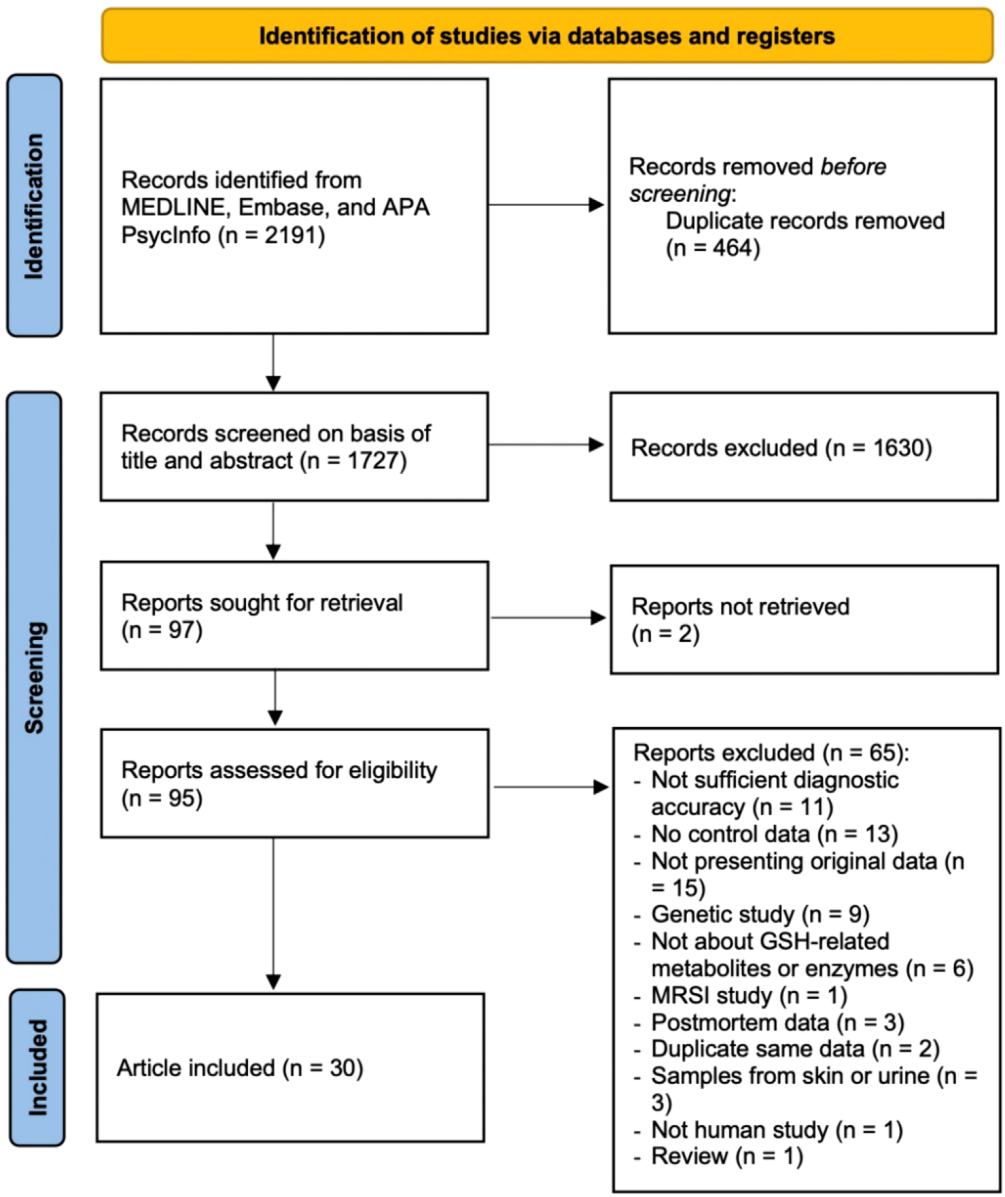
Preferred reporting items for systematic reviews and meta‐analysis (PRISMA) diagram for study search. APA, American Psychological Association; Embase, Excerpta Medica Database; MEDLINE, Medical Literature Analysis and Retrieval System On‐Line; PRISMA, Preferred Reporting Items for Systematic Reviews and Meta‐Analyses.

Out of 2191 initial records, a total of 30 studies involving 1966 participants (*n* = 1019 for the patient group; *n* = 947 for the HC group) met the eligibility criteria (Figure 2), with their characteristics summarized in Table 1.

**TABLE 1:** Characteristics of studies included for meta‐analysis.

Author year	*N*	HC			Patients with depression							
		Age (SD) (years)	Proportion of males (%)	N	Age (SD) (years)	Proportion of males (%)	Medication	Proportion of medicated patients (%)	Duration of illness (months)	HAMD‐17	Materials	Metabolites and enzymes
Bilici et al. (2001)	32	42.1 (7.4)	50	30	41.5 (NA)	30	No	0	75.6	24.1	Plasma	GPx, GR
											Erythrocyte	GPx, GR
Camkurt et al. (2016)	59	30.51 (5.68)	0	45	29.04 (5.46)	0	No	0	NA	NA	Umbilical blood	GPx
Cimen et al. (2015)	18	37.89 (10.62)	44.44	18	42.17 (10.16)	38.89	No	0	3.93	NA	Eryghrocyte	GPx
Diniz et al. (2018)	47	70.1 (7.1)	6.38	77	72.6 (7.7)	14.29	NA	NA	NA	NA	Plasma	GPx, GR, GST
Draganov et al. (2020)	63	41.77 (10.1)	46.88	18	NA	NA	No	0	NA	23.44	MRS	GSH
Freed et al. (2017)	8	16.1 (3.4)	37.5	19	15.7 (2.5)	57.89	No	0	26.9	NA	MRS	GSH
Galecki et al. (2009)	30	32.1 (4.3)	46.67	50	36.7 (5.2)	44	No	0	NA	NA	Erythrocyte	GPx
Godlewska et al. (2015)	27	30.3 (10.6)	59.3	33	29.9 (10.6)	57.58	No	0	NA	22.3	MRS	GSH
Hermens et al. (2018)	59	23.8 (2.9)	35.59	94	21.5 (2.8)	42.55	Yes	62	64.8	13.7	MRS	GSH
Jollant et al. (2017)	33	31.2 (6.5)	39.39	25	40.2 (NA)	40	No	0	85.4	NA	MRS	GSH
Jordan et al. (2018)	43	35.1 (4.6)	60.47	18	45.0 (5.0)	61.11	No	0	NA	NA	Serum	GST
Kaddurah‐Daouk et al. (2012)	18	40.0 (11.0)	44	28	41.5 (NA)	52	No	0	252	9.3	CSF	GSH
Kodydkova et al. (2009)	35	65.1 (6.0)	0	35	64.0 (6.1)	0	NA	NA	NA	NA	Blood	GSH
											Erythrocyte	GPx, GR
Kotan et al. (2011)	44	33.2 (7.9)	22.73	50	33.1 (10.0)	22	No	0	NA	30.4	Serum	GPx
Lackovic et al. (2023)	32	45.4 (11.4)	41	32	51.1 (9.6)	28	NA	NA	137.28	NA	Serum	GPx, GR
Lindqvist et al. (2014)	19	36.5 (12.1)	36.84	16	33.6 (7.2)	37.5	No	0	NA	19.05	Blood	GSH, GPx
Mahdi et al. (2022)	30	NA	NA	30	NA	NA	NA	NA	NA	NA	Blood	GPx, GR
Oglodek (2017)	40	42.4 (4.1)	50	180	45.27 (NA)	50	No	0	6	NA	Serum	GR
Oglodek et al. (2017)	40	42.4 (4.1)	50	180	45.27 (NA)	50	No	0	6	NA	Serum	GPx
Ormonde do Carmo et al. (2015)	27	33.0 (2.0)	37.04	22	31.0 (2.0)	18.18	No	0	NA	NA	Serum	GPx
											Platelets	GPx
Rybka et al. (2013)	19	62.3 (2.8)	NA	15	59.7 (1.9)	NA	Yes	100	NA	NA	Blood	GSH
											Erythrocyte	GPx, GR
Samaryn et al. (2023)	30	45.4 (NA)	33	33	41.4 (NA)	40	Yes	63.6	NA	NA	Serum	GSH, GPx
Sarandol et al. (2007)	54	37.0 (9.0)	25.93	96	40.0 (11.0)	25	No	0	NA	NA	Blood	GPx
Shungu (2012)	13	27.6 (7.4)	46.15	15	31.7 (9.6)	40	No	0	105.6	NA	MRS	GSH
Silva et al. (2019)	20	58.2 (8.5)	65	16	59.2 (7.1)	56.25	Yes	100	84	NA	Blood	GSH
Smith et al. (2021)	9	67.0 (7.0)	55.56	9	70.0 (7.0)	44.44	No	0	NA	NA	MRS	GSH
Srivastava et al. (2002)	18	NA	NA	12	NA	NA	No	0	NA	NA	Polymorphonuclear leukocyte	GPx
Stefanescu et al. (2012)	20	46.3 (7.8)	35	31	47.5 (NA)	35.48	Yes	100	85.2	NA	Blood	GPx
Tsai and Huang et al. (2016)	40	33.0 (5.7)	25	21	49.6 (7.0)	19.05	No	0	NA	27.4	Serum	GPx
Tuura et al. (2023)	20	27.5 (5.2)	37	12	22.3 (2.8)	42	No	0	NA	NA	MRS	GSH

Fourteen articles were identified for the studies included in the meta‐analyses of GSH comprising an overall sample of 367 patients and 373 HC. The comparison in GSH between the two groups was reported in 14 studies. The average number of patients and HC in each study was 26.21 ± 20.61 (range: 9–94) and 26.64 ± 16.07 (range: 8–63), respectively. Average age and percentage of males in patient and HC groups were 40.18 ± 16.07 and 41.02 ± 16.06 years and 41.65% ± 13.82% and 39.85% ± 14.30%, respectively. Materials used in studies and their number were as follows: four for whole blood, one for serum, one for CSF, and eight for ^1^H‐MRS. One article was identified for the meta‐analysis of GSSG (*n* = 16 for patients and *n* = 19 for HC). Averages for the number of patients and HC, as well as age and the percentage of males in the patient and HC groups, were not calculated due to the inclusion of only one study. Material used in the study and the number were as follows: for GSSG study, whole blood (*k* = 1).

Among the 14 GSH studies, two included two independent comparisons, resulting in a total of 16 samples. Hereinafter, *k* refers to the number of independent comparisons; therefore, in this case, *k* = 16. Among the 14 GSH studies (*k* = 16), four studies [27, 28, 29, 30] and two studies [31, 32] showed significantly decreased and increased GSH levels, respectively, in patients with depression compared to HC, while 8 studies (*k* = 10) did not report any significant difference between the two groups [33, 34, 35, 36, 37, 38, 39, 40]. The GSSG study [37] reported no significant difference between the two groups.

Eighteen (*k* = 20), eight (*k* = 9), and two (*k* = 2) articles were identified for the meta‐analyses of GPx (*n* = 673 for patients and *n* = 594 for HC), (*n* = 293 for patients and *n* = 253 for HC), and GST (*n* = 95 for patients and *n* = 90 for HC), respectively. Materials and the number of comparisons were as follows: for GPx studies, whole blood (*k* = 5), erythrocyte (*k* = 5), serum (*k* = 6), plasma (*k* = 2), polymorphonuclear leukocyte (*k* = 1), and platelet (*k* = 1). For GR studies, whole blood (*k* = 1), erythrocyte (*k* = 3), serum (*k* = 2), plasma (*k* = 2), and CSF (*k* = 1). For GST studies, serum (*k* = 1), and plasma (*k* = 1). We found no articles providing the data of GCL or GS.

Among the 18 GPx studies (*k* = 20), eight studies (*k* = 8) showed significantly decreased GPx activity [29, 31, 41, 42, 43, 44, 45, 46] and one (*k* = 1) showed increased GPx activity [47] in patients with depression compared to HC, while nine studies (*k* = 11) did not report any significant difference between the two groups [37, 38, 48, 49, 50, 51, 52, 53, 54]. Among the eight GR studies (*k* = 9), four studies (*k* = 5) showed significantly increased GR activity in patients with depression compared to HC [29, 31, 44, 47, 55] and one (*k* = 1) showed decreased GR activity [43], while three studies (*k* = 3) did not report any significant difference between the two groups [36, 42, 47]. Among the two GST studies, both studies (*k* = 2) showed no significant difference between the two groups [42, 56].

The Newcastle‐Ottawa Scale score ranged from 3 to 6 and the average was 5.5 (Table S1), which suggests that the quality of the included studies was good on average.

### 3.2 Meta‐Analysis

#### 3.2.1 The Primary Meta‐Analysis

**FIGURE 3:**
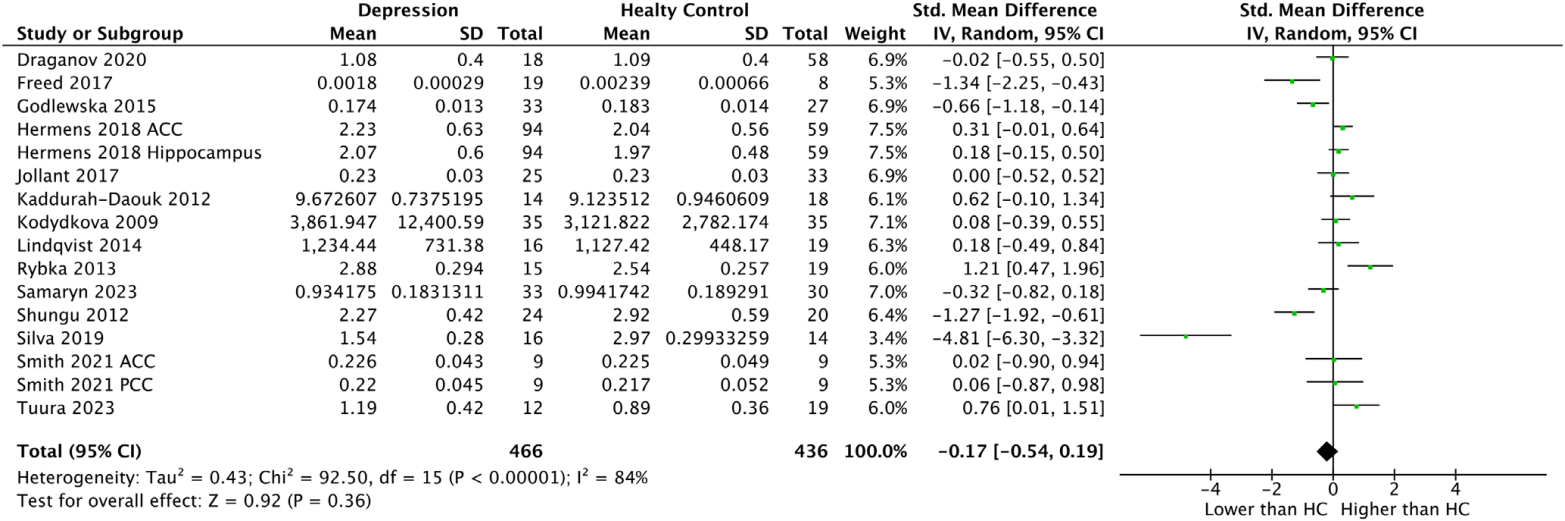
Forest plot of random‐effects meta‐analysis comparing the level of GSH in whole samples between patients with depression and HC. CI, confidence interval; df, degree of freedom; GSH, glutathione; HC, healthy controls; SD, standard deviation; std., standardized.

In GSH levels of whole sample (*k* = 16, SMD = −0.17, CI = −0.54–0.19, *p* = 0.36, *I* ^2^ = 84%) (Figure 3), central sample (*k* = 11, SMD = −0.10, CI = −0.44–0.25, *p* = 0.58, *I* ^2^ = 75%) (Figure S1) and peripheral sample (*k* = 5, SMD = −0.42, CI = −1.46–0.63, *p* = 0.43, *I* ^2^ = 92%) (Figure S2), no significant differences were detected between patients with depression and HC. With regard to GSSG samples, SMDs could not be calculated due to the small number of included studies (*k* = 1).

#### 3.2.2 The Secondary Meta‐Analysis

**FIGURE 4:**
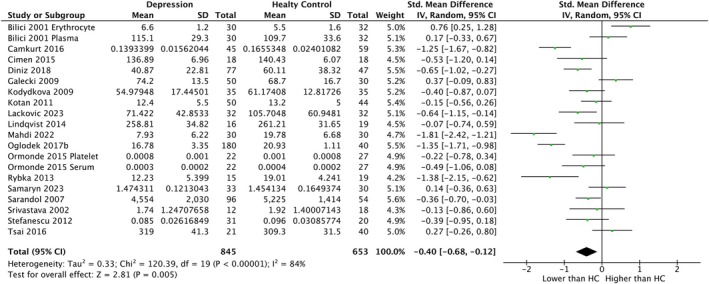
Forest plot of random‐effects meta‐analysis comparing the activity of GPx in whole samples between patients with depression and HC. CI, confidence interval; df, degree of freedom; GPx, glutathione peroxidase; HC, healthy controls; SD, standard deviation; std., standardized.

**FIGURE 5:**
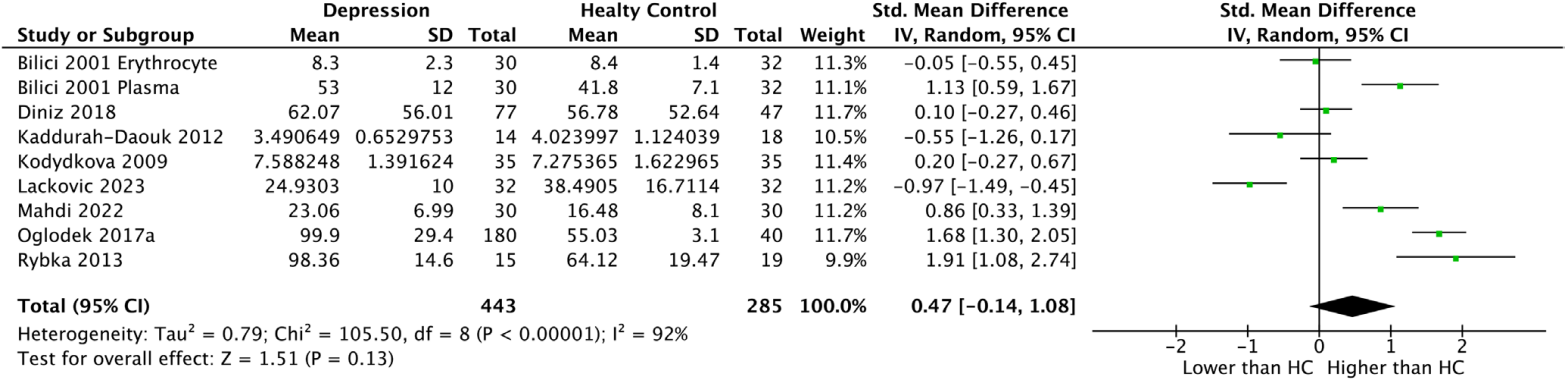
Forest plot of random‐effects meta‐analysis comparing the activity of GR in whole samples between patients with depression and HC. CI, confidence interval; df, degree of freedom; GR, glutathione reductase; HC, healthy controls; SD, standard deviation; std., standardized.

In the whole sample, GPx activity was decreased in patients with depression with small SMD (*k* = 20, SMD = −0.40, CI = −0.68 to −0.12, *p* = 0.005, *I* ^2^ = 84%) (Figure 4). For GR levels, no significant difference was found between patients with depression and HC (*k* = 9, SMD = 0.47, CI = −0.14–1.08, *p* = 0.13, *I* ^2^ = 92%) (Figure 5). With regard to GST samples, SMDs could not be calculated due to the small number of the included studies (*k* = 2). For GR levels in the peripheral sample, no significant difference was found between patients with depression and HC (*k* = 8, SMD = 0.59, CI = −0.05–1.23, *p* = 0.07, *I* ^2^ = 93%) (Figure S8). For GPx activity, we did not perform meta‐analyses for central and peripheral samples because all the included studies were for peripheral samples. With regard to GST activity in the central sample, SMDs could not be calculated due to the small number of the included studies (*k* = 1).

### 3.3 Subgroup Analysis and Sensitivity Analysis

#### 3.3.1 Materials

GSH levels in MRS (*k* = 10, SMD = −0.16, CI = −0.52 to 0.20, *p* = 0.38, *I* ^2^ = 76%) (Figure S3) and whole blood (*k* = 4, SMD = −0.66, CI = −2.15–0.84, *p* = 0.39, *I* ^2^ = 94%) (Figure S4) samples showed no significant differences between patients with depression and HC. GSH levels in serum (*k* = 1) and CSF (*k* = 1) could not be calculated due to the limited number of studies.

No significant SMD of GPx activities was detected between patients with depression and HC in the whole blood (*k* = 5, SMD = −0.78, CI = −1.36 to −0.19, *p* = 0.009 > 0.00625, *I* ^2^ = 86%) (Figure S5), serum (*k* = 6, SMD = −0.38, CI = −0.91–0.15, *p* = 0.16, *I* ^2^ = 87%) (Figure S6), and erythrocyte samples (*k* = 5, SMD = −0.20, CI = −0.86–0.47, *p* = 0.56, *I* ^2^ = 86%) (Figure S7). GR activity of the erythrocyte (*k* = 3, SMD = 0.62, CI = −0.33 to 1.58, *p* = 0.20, *I* ^2^ = 88%) showed no significant difference between the two groups (Figure S9).

GPx activity in the plasma (*k* = 2), platelet (*k* = 1), and polymorphonuclear samples (*k* = 1), GR levels in the whole blood (*k* = 1), plasma (*k* = 2), serum (*k* = 2), and CSF (*k* = 1) samples, GSSG level in the whole blood (*k* = 1), and GST activities in plasma (*k* = 1) and serum (*k* = 2) samples; SMD could not be calculated due to the limited number of published papers.

#### 3.3.2 Medication

GSH levels and GPx levels in the whole samples were investigated on the intake of medications. No significant difference was found in GSH level between medicated patients with depression and HC (*k* = 5, SMD = −0.36, CI = −1.19 to 0.47, *p* = 0.39, *I* ^2^ = 93%), or between unmedicated patients and HC (*k* = 8, SMD = −0.21, CI = −0.69 to 0.28, *p* = 0.41, *I* ^2^ = 79%) (Figure S10). No significant difference was found in GPx activity between medicated patients with depression and HC (*k* = 3, SMD = −0.50, CI = −1.30 to 0.31, *p* = 0.22, *I* ^2^ = 82%), or between unmedicated patients and HC (*k* = 10, SMD = −0.28, CI = −0.66 to 0.10, *p* = 0.14, I^2^ = 86%) (Figure S11). We could not perform subgroup analyses for GSSG, GR, or GST due to the small number of studies.

#### 3.3.3 Sensitivity Analysis

Leave‐one‐out sensitivity analysis showed that the results of whole GPx, which showed a significant decrease in patients with depression, were robust. The result of GPx in the whole blood changed when leaving out Lindqvist et al. [37] (*k* = 4, SMD = −0.94, CI = −1.59 to −0.28, *p* = 0.005, *I* ^2^ = 90%), showing a decreased activity in patients with depression compared with HC.

### 3.4 Meta‐Regression Analysis

We found no significant relationship between the SMDs of the outcomes and modulators for GSH, GPx, and GR (Table S2–S4).

### 3.5 Publication Bias

Egger's test suggested publication bias for GSH (*p* = 0.0034), and not for GPx (*p* = 0.86) and GR (*p* = 0.75). The funnel plots are displayed in Figure S12. We did not perform Egger's test for GSSG and GST due to the small number of studies (*k* = 1, and *k* = 2, respectively). Because publication bias for GSH was suggested, we conducted a trim‐and‐fill analysis. However, the analysis did not add any publication, and the result of the meta‐analysis did not change.

## 4 Discussion

### 4.1 Main Findings

This is the first meta‐analysis to comprehensively compare GSH‐related metabolite levels and enzyme activities between patients with depression and HC, including data from various sample sources and measurement methods. Our main findings are twofold: (1) the activity of GPx was lower in patients with depression compared with HC with a small effect size; (2) there was no significant difference in the level of GSH between the two groups. Regarding GSSG level, although GSSG was one of our primary foci, we could not perform a meta‐analysis for GSSG due to the small number of studies.

The strengths of our study are as follows. First, this is the first meta‐analysis comprehensively comparing GSH‐related metabolite levels and enzyme activities between patients with depression and HC. Second, we incorporated nearly twice as many studies as the most recent meta‐analysis on GPx activity [18], thereby updating the findings and addressing inconsistencies in previous meta‐analyses. Lastly, we conducted moderator analyses to examine potential factors influencing GSH‐related metabolite levels and enzyme activities, focusing on medication status, age, proportion of males, proportion of medicated patients, duration of illness, and severity of illness.

### 4.2 Primary Outcome

#### 4.2.1 Glutathione

Our findings did not demonstrate a significant difference in GSH levels between patients with depression and HC for the whole, central, and peripheral samples. While four out of 14 studies indicated reduced GSH levels in patients with depression compared to HC [27, 28, 29, 30], the majority of the studies, eight in total, reported no significant differences [33, 34, 35, 36, 37, 38, 39, 40]. Therefore, consistent with the majority of these studies, our results suggest that GSH levels do not significantly deviate in patients with depression, indicating that GSH levels per se may not contribute to the pathophysiology of depression. While studies utilizing animal models with stress‐induced depression and rodent models of depression [57, 58] have reported decreased GSH levels in these models, and a postmortem study [59] showed lower GSH levels in patients with depression, our results did not corroborate these findings.

One possible reason for the inconsistencies between our findings and studies that report a significant reduction in GSH levels in patients with depression could be that a decrease in GSH may only be observed in certain brain regions such as the occipital cortex [27, 28, 30] and prefrontal cortex [59], but not in other regions such as the ACC [34, 40]. This interpretation is further supported by a recent meta‐analysis [16], which reported decreased GSH levels exclusively in the occipital cortex. Furthermore, three out of the five studies that measured GSH levels in blood samples reported no significant differences between the groups [37, 38, 39], suggesting that GSH levels in peripheral samples may not differ between patients with depression and HC. To confirm this with a meta‐analysis examining GSH levels for each brain region, we would require further research, given the limited number of existing studies.

In our subgroup analyses, we found that medications had no effect on GSH levels. Among previous studies reporting a significant reduction in GSH levels in patients with depression, two focused on patients not currently on medication [27, 28], and one considered patients who had been medication‐free for over 2 weeks [30]. These studies indicate a reduction in GSH levels among medication‐free patients. Furthermore, a previous study on mice reported increased GSH levels after the administration of selective serotonin reuptake inhibitors (SSRIs) (sertraline, fluvoxamine) [60]. As such, we anticipated that patients off medication would exhibit a decrease in GSH levels. However, our results did not find such results.

#### 4.2.2 GSH Disulfide

Regarding GSSG, a meta‐analysis could not be performed due to the limited number of studies available (*k* = 1). While this study reported no significant difference in GSSG levels between patients with depression and HC [37], further research is needed to explore the relationship between GSSG levels and the pathophysiology of depression.

### 4.3 Secondary Outcomes

#### 4.3.1 Glutathione‐Peroxidase

Across the whole patient sample, our study identified reduced GPx activity in patients with depression when compared with HC. This finding aligns with the most recent meta‐analysis on GPx activity, which also reported a decrease in GPx activity in patients with depression [18]. However, our results did not align with two other meta‐analyses that found no significant differences in GPx activity levels between the two groups [13, 17]. This inconsistency is likely due to the disparity in the number of studies included in the respective meta‐analyses. Two previous meta‐analyses that reported no significant group differences included seven and six studies, respectively. In contrast, the most recent meta‐analysis, which reported findings consistent with the present study, included 14 studies. Our meta‐analysis incorporated 18 studies (*k* = 20), thereby supporting the findings of the most recent study and further demonstrating the robustness of the reduced GPx activity in patients with depression compared to HC, by adding four additional studies. Our findings also align with those of an animal study that demonstrated an increase in GPx activity by the intake of escitalopram (an SSRI) and lamotrigine [61]. However, it is important to note that the heterogeneity among the studies was as high as *I* ^2^ of 84%, which is consistent with the most recent meta‐analysis showing a similar value of 87% [18]. Several factors may have contributed to this. First, we included studies that measured GPx activity in different sample types, including whole blood, serum, plasma, and erythrocytes. Second, there was variability in patients' treatment conditions; some patients were on medication, others had undergone electroconvulsive therapy (ECT), and some were treatment‐naïve.

In our subgroup analyses, we found no significant difference in GPx activity between patients with depression and HC in the whole blood, serum, or erythrocyte samples. In terms of medication status, we found no significant impact on GPx activity, a result that aligns with a prior meta‐analysis that reported no significant difference in GPx activity before and after antidepressant treatment [17].

In our meta‐regression analyses, no significant impact of any factors (i.e., age, proportion of males, proportion of medicated patients, duration of illness, and severity of illness) was found. An animal study demonstrated an increase in GPx activity following the intake of escitalopram (an SSRI) and lamotrigine [61]. Regarding the severity of the illness, previous studies have reported inconsistent conclusions. While several studies found a negative correlation between GPx activity and the severity of depression [41, 42], others reported no significant relationship between the two [29, 48, 54]. Our conclusions must be tempered due to focusing solely on the HAMD‐17 score and the limited number of studies included. Nonetheless, our results suggest that although GPx activity is reduced in patients with depression, the degree of this reduction does not necessarily correspond to the severity of depression.

Our results provide significant insights into how GSH‐related metabolites and enzymes may be associated with the pathophysiology of depression. Employing GSH as a cofactor, GPx plays a pivotal role in reducing toxic oxidants, including hydrogen peroxide [15]. Therefore, in patients with depression, diminished GPx activity likely leads to elevated levels of these toxic oxidants, triggering inflammation, which contributes to the pathophysiology of depression [62]. Although we did not investigate the level of H_2_O_2_ in patients with depression in our study, an inverse correlation between the levels of GPx and H_2_O_2_ has been reported [31]. This supports the idea that decreased GPx activity leads to a rise in H_2_O_2_ levels, contributing to inflammation in depression. In support, there is evidence reporting central (e.g., increased 17‐kDA translocator protein binding) and peripheral (e.g., increased CRP levels, and higher neutrophil and monocyte counts) inflammation in patients with depression [63, 64].

#### 4.3.2 GSH Reductase

We found no significant difference in GR activity between patients with depression and HC, which is consistent with the result of a previous meta‐analysis [13]. GR is an enzyme that reduces GSSG to GSH and is up‐regulated under conditions of oxidative stress [65, 66]. Indeed, four out of eight studies included in our meta‐analysis reported increased GR activity in patients with depression [29, 31, 44, 47, 55]. However, the results of the meta‐analysis did not support this finding. Since a previous meta‐analysis that included three studies using red blood cell samples also reported no significant differences between patients with depression and HC [13], our results support and extend these findings by incorporating additional sample types.

In our subgroup analyses, we found no significant effect of factors including medication status, age, or proportion of males. While most studies did not explore the effects of these factors, a previous study reported significantly higher GR activity in female patients compared to males across mild, moderate, and severe depression [55]. Therefore, it is plausible that there may be a sex‐related difference concerning the extent of alteration in GR activity in patients with depression, especially increased activity in female patients. Further research is needed to elucidate the effect of sex more in detail.

#### 4.3.3 GSH S‐Transferase

Regarding GST, a meta‐analysis was infeasible due to the limited number of studies (*k* = 2). Because both studies found no significant difference in GST activity between the depression and HC groups [42, 56], GST activity might not be relevant to the pathophysiology of depression, nor serve as a viable biomarker for depression, while definitive conclusions cannot be drawn at this point.

#### 4.3.4 GCL and GS

Regarding GCL and GS, we found no studies that compared their activities between patients with depression and HC. Our findings suggest that, in order to gain a comprehensive understanding of the GSH cycle in depression, future studies are needed to investigate and compare the activities of these enzymes between the two groups.

### 4.4 Relationship Between GSH Level and GPx Activity

Finally, we provide a comprehensive discussion on the results regarding the potential pathophysiology of depression based on our result of abnormal GSH cycling. In the current study, we found a decreased GPx activity in patients with depression compared with HC, while no difference was found in GSH levels between the two groups. As discussed earlier, the reduction of GPx activity, which contributes to an increase in the level of H_2_O_2_, is assumed to trigger inflammation in patients with depression. This result aids in interpreting the non‐significant difference in GSH levels between patients with depression and HC. Presumably, because GPx activity is reduced in patients with depression, GSH levels remain relatively stable as GPx can no longer effectively utilize GSH as a cofactor. This further suggests that inflammation stemming from decreased GPx activity contributes to the pathophysiology of depression, rather than inflammation being a consequence of depression, because if the latter were true, GSH levels should also decrease alongside the reduction in GPx.

### 4.5 Limitations

This study has several limitations. First, the methods employed for metabolite examination varied across studies, ranging from MRS to whole blood analysis. Second, this research did not account for confounding factors such as smoking habits or dietary patterns, even though these elements have been reported to impact GSH levels in both humans and rats [67, 68, 69]. Third, due to the paucity of available studies, we were unable to perform meta‐analyses on several metabolites such as GSSG and GST. Fourth, the limited number of studies also prevented us from conducting several subgroup analyses. Lastly, heterogeneity among the studies was high for GPx activity (*I* ^2^ = 84%), in which we found a significant difference between patients with depression and HC. Several factors may have contributed to this, including differences in the sample types measured and variability in patients' treatment conditions.

## 5 Conclusion

This is the first meta‐analysis to comprehensively compare GSH‐related metabolite levels and enzyme activities (i.e., GSH, GSSG, GPx, GR, GCL, GS, and GST) between patients with depression and HC, including data from various sample sources and measurement methods. This meta‐analysis revealed decreased GPx activity in patients with depression compared with HC, while no significant differences in the levels of GSH were found between the two groups. Our findings suggest that abnormal cycling of the GSH system contributes to the pathophysiology of depression, and that decreased GPx activity, in particular, could potentially serve as a biomarker for the disease. In addition, our results suggest that increasing GPx activity with N‐acetylcysteine, glutathione, cysteine, or selenium may potentially be effective for depression by targeting the GSH system as indicated by a previous study [70]. Investigating whether they are effective in treating depression by conducting prospective studies is one of the important future directions. However, although we conducted meta‐regression analyses on GPx activity, which had not been performed in previous meta‐analyses, we did not identify any significant moderators associated with GPx activity. In addition, although we aimed to provide a comprehensive overview of the GSH cycle by including GSH‐related metabolites and enzyme activities not covered in previous meta‐analyses, we were unable to perform meta‐analyses on GSSG, GCL, GS, and GST due to the limited number of studies comparing their levels or activities between patients with depression and HC. Overall, our findings highlight the need for further research on these metabolites and enzymes to gain a more detailed understanding of the GSH cycle in depression.

## Funding

This study was supported by the Japan Society for the Promotion of Science (18H02755, 22H03002), Japan Agency for Medical Research and Development (AMED: JP24wm0625302), Japan Research Foundation for Clinical Pharmacology, Naito Foundation, Watanabe Foundation, and Takeda Science Foundation.

## Ethics Statement

The authors have nothing to report.

## Conflicts of Interest

The authors declare no conflicts of interest.

## Supporting information


**Figure S1:** Forest plot of random‐effects meta‐analysis comparing the level of GSH for central samples between patients with depression and HC.
**Figure S2:** Forest plot of random‐effects meta‐analysis comparing the level of GSH for peripheral samples between patients with depression and HC.
**Figure S3:** Forest plot of random‐effects meta‐analysis comparing the level of GSH for MRS samples between patients with depression and HC.
**Figure S4:** Forest plot of random‐effects meta‐analysis comparing the level of GSH for whole blood samples between patients with depression and HC.
**Figure S5:** Forest plot of random‐effects meta‐analysis comparing the level of GPx for whole blood samples between patients with depression and HC.
**Figure S6:** Forest plot of random‐effects meta‐analysis comparing the level of GPx for serum samples between patients with depression and HC.
**Figure S7:** Forest plot of random‐effects meta‐analysis comparing the level of GPx for erythrocyte samples between patients with depression and HC.
**Figure S8:** Forest plot of random‐effects meta‐analysis comparing the level of GR for peripheral samples between patients with depression and HC.
**Figure S9:** Forest plot of random‐effects meta‐analysis comparing the level of GR for erythrocyte samples between patients with depression and HC.
**Figure S10:** Forest plot of random‐effects meta‐analysis comparing the level of GSH in whole samples between medicated and unmedicated patients with depression and HC.
**Figure S11:** Forest plot of random‐effects meta‐analysis comparing the level of GPx in whole samples between medicated and unmedicated patients with depression and HC.
**Figure S12:** Funnel plots of GSH‐related metabolites in whole samples.
**Table S1:** Summary of overall quality of evidence assessed using Newcastle‐Ottawa Quality Assessment Scale.
**Table S2:** Results of univariate meta‐regression analyses on GSH in whole samples.
**Table S3:** Results of univariate meta‐regression analyses on GPx in whole samples.
**Table S4:** Results of univariate meta‐regression analyses on GR in whole samples.

## Data Availability

The data that support the findings of this study are openly available in the Open Science Framework (OSF) at https://osf.io/6f3kz/overview?view_only=690eb99ccd0e464ca9115e9e8e45d302.
