# Supplementary material for: Glutathione‐Related Metabolite Levels and Enzyme Activities in Depression: A Systematic Review and Meta‐Analysis
Source: Neuropsychopharmacol Rep. 2026 Apr 30;46(2):e70125. doi: 10.1002/npr2.70125 (PMC13129703; doi:10.1002/npr2.70125)
Supplement: Supplementary file 1 — Figure S1: Forest plot of random‐effects meta‐analysis comparing the level of GSH for central samples between patients with depression and HC. Figure S2: Forest plot of random‐effects meta‐analysis comparing the level of GSH for peripheral samples between patients with depression and HC. Figure S3: Forest plot of random‐effects meta‐analysis comparing the level of GSH for MRS samples between patients with depression and HC. Figure S4: Forest plot of random‐effects meta‐analysis comparing the level of GSH for whole blood samples between patients with depression and HC. Figure S5: Forest plot of random‐effects meta‐analysis comparing the level of GPx for whole blood samples between patients with depression and HC. Figure S6: Forest plot of random‐effects meta‐analysis comparing the level of GPx for serum samples between patients with depression and HC. Figure S7: Forest plot of random‐effects meta‐analysis comparing the level of GPx for erythrocyte samples between patients with depression and HC. Figure S8: Forest plot of random‐effects meta‐analysis comparing the level of GR for peripheral samples between patients with depression and HC. Figure S9: Forest plot of random‐effects meta‐analysis comparing the level of GR for erythrocyte samples between patients with depression and HC. Figure S10: Forest plot of random‐effects meta‐analysis comparing the level of GSH in whole samples between medicated and unmedicated patients with depression and HC. Figure S11: Forest plot of random‐effects meta‐analysis comparing the level of GPx in whole samples between medicated and unmedicated patients with depression and HC. Figure S12: Funnel plots of GSH‐related metabolites in whole samples. Table S1: Summary of overall quality of evidence assessed using Newcastle‐Ottawa Quality Assessment Scale. Table S2: Results of univariate meta‐regression analyses on GSH in whole samples. Table S3: Results of univariate meta‐regression analyses on GPx in whole samples. Table S4: Results of uni [file NPR2-46-e70125-s001.docx]

**Supplementary Materials**

**Supplementary Figures Captions**

**Supplementary Figure 1**

| **** |
| --- |
| Supplementary Figure 1: Forest plot of random-effects meta-analysis comparing the level of GSH for central samples between patients with depression and HC.  Abbreviations: CI, confidence interval; df, degree of freedom; GSH, glutathione; HC, healthy controls; SD, standard deviation; std, standardized |

**Supplementary Figure 2**

| **** |
| --- |
| Supplementary Figure 2: Forest plot of random-effects meta-analysis comparing the level of GSH for peripheral samples between patients with depression and HC.  Abbreviations: CI, confidence interval; df, degree of freedom; GSH, glutathione; HC, healthy controls; SD, standard deviation; std, standardized |

**Supplementary Figure 3**

| **** |
| --- |
| Supplementary Figure 3: Forest plot of random-effects meta-analysis comparing the level of GSH for MRS samples between patients with depression and HC.  Abbreviations: CI, confidence interval; df, degree of freedom; GSH, glutathione; HC, healthy controls; MRS, magnetic resonance spectroscopy; SD, standard deviation; std, standardized |

**Supplementary Figure 4**

| **** |
| --- |
| Supplementary Figure 4: Forest plot of random-effects meta-analysis comparing the level of GSH for whole blood samples between patients with depression and HC.  Abbreviations: CI, confidence interval; df, degree of freedom; GSH, glutathione; HC, healthy controls; SD, standard deviation; std, standardized |

**Supplementary Figure 5**

| **** |
| --- |
| Supplementary Figure 5: Forest plot of random-effects meta-analysis comparing the level of GPx for whole blood samples between patients with depression and HC.  Abbreviations: CI, confidence interval; df, degree of freedom; GPx, glutathione peroxidase; HC, healthy controls; SD, standard deviation; std, standardized |

**Supplementary Figure 6**

| **** |
| --- |
| Supplementary Figure 6: Forest plot of random-effects meta-analysis comparing the level of GPx for serum samples between patients with depression and HC.  Abbreviations: CI, confidence interval; df, degree of freedom; GPx, glutathione peroxidase; HC, healthy controls; SD, standard deviation; std, standardized |

**Supplementary Figure 7**

| **** |
| --- |
| Supplementary Figure 7: Forest plot of random-effects meta-analysis comparing the level of GPx for erythrocyte samples between patients with depression and HC.  Abbreviations: CI, confidence interval; df, degree of freedom; GPx, glutathione peroxidase; HC, healthy controls; SD, standard deviation; std, standardized |

**Supplementary Figure 8**

| **** |
| --- |
| Supplementary Figure 8: Forest plot of random-effects meta-analysis comparing the level of GR for peripheral samples between patients with depression and HC.  Abbreviations: CI, confidence interval; df, degree of freedom; GR, glutathione reductase; HC, healthy controls; SD, standard deviation; std, standardized |

**Supplementary Figure 9**

| **** |
| --- |
| Supplementary Figure 9: Forest plot of random-effects meta-analysis comparing the level of GR for erythrocyte samples between patients with depression and HC.  Abbreviations: CI, confidence interval; df, degree of freedom; GR, glutathione reductase; HC, healthy controls; SD, standard deviation; std, standardized |

**Supplementary Figure 10**

| **** |
| --- |
| Supplementary Figure 10: Forest plot of random-effects meta-analysis comparing the level of GSH in whole samples between medicated and unmedicated patients with depression and HC.  Abbreviations: CI, confidence interval; df, degree of freedom; GSH, glutathione; HC, healthy controls; SD, standard deviation; std, standardized |

**Supplementary Figure 11**

| **** |
| --- |
| Supplementary Figure 11: Forest plot of random-effects meta-analysis comparing the level of GPx in whole samples between medicated and unmedicated patients with depression and HC.  Abbreviations: CI, confidence interval; df, degree of freedom; GPx, glutathione peroxidase; HC, healthy controls; SD, standard deviation; std, standardized |

**Supplementary Figure 12**

(A)

| **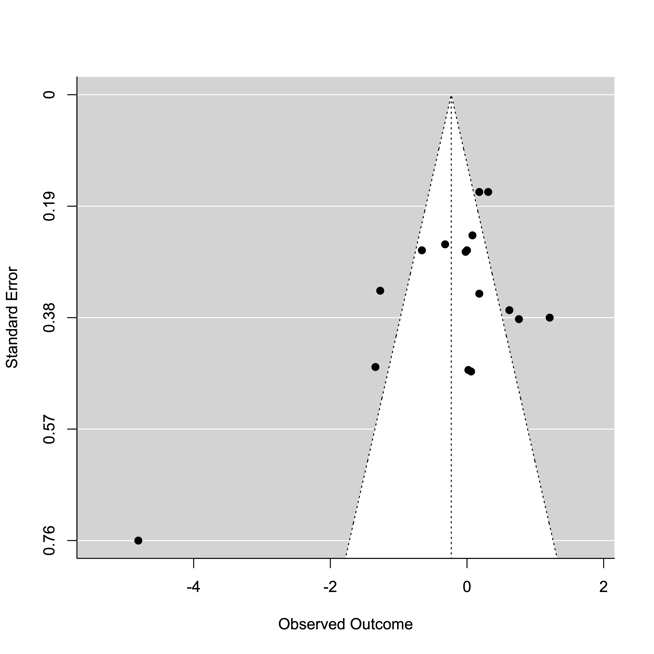**  (B) | **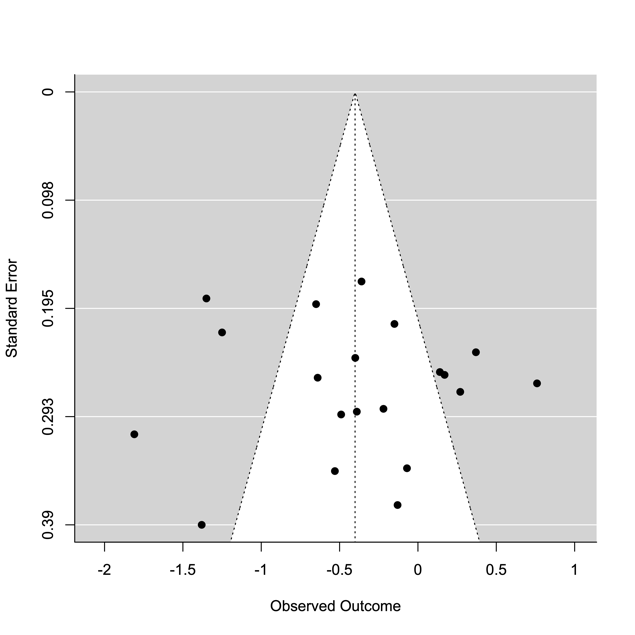**  (C) | **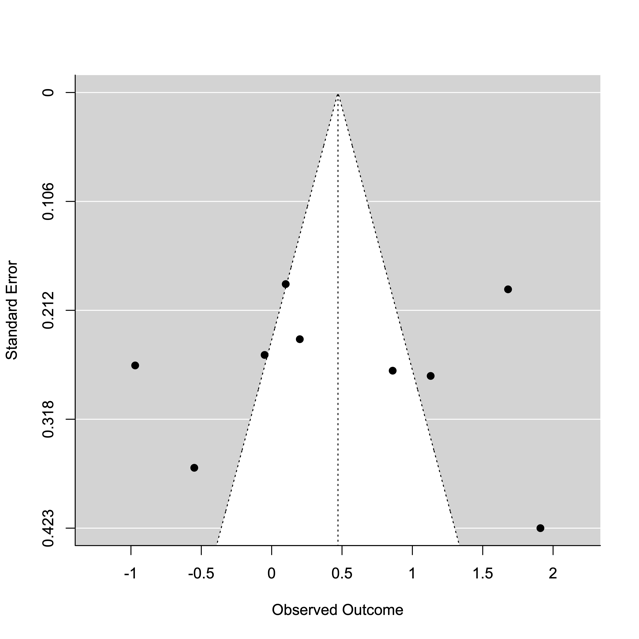** |
| --- | --- | --- |
| Supplementary Figure 12: Funnel plots of GSH-related metabolites in whole samples.  (A) GSH  Abbreviations: GSH, glutathione  (B) GPx  Abbreviations: GPx, glutathione peroxidase  (C) GR  Abbreviations: GR, glutathione reductase | | |

**Supplementary Tables**

Supplementary Table 1: Summary of overall quality of evidence assessed using Newcastle-Ottawa Quality Assessment Scale.

| 1st Author (Year) | Selection | Comparability | Total |
| --- | --- | --- | --- |
| Bilici et al. (2001) | ☆☆☆☆ | ☆☆ | 6 |
| Camkurt et al. (2016) | ☆☆☆☆ | ☆☆ | 6 |
| Cimen et al. (2015) | ☆☆☆☆ | ☆☆ | 6 |
| Diniz et al. (2018) | ☆☆☆☆ | ☆☆ | 6 |
| Draganov et al. (2020) | ☆☆☆☆ | ☆☆ | 6 |
| Freed et al. (2017) | ☆☆☆☆ | ☆☆ | 6 |
| Galecki et al. (2009) | ☆☆☆☆ | ☆☆ | 6 |
| Godlewska et al. (2015) | ☆☆☆☆ | ☆☆ | 6 |
| Hermens et al. (2018) | ☆☆☆☆ | ☆ | 5 |
| Jollant et al. (2017) | ☆☆☆☆ | ☆ | 5 |
| Jordan et al. (2018) | ☆☆☆☆ | ☆☆ | 6 |
| Kaddurah-Daouk et al. (2012) | ☆☆☆☆ | ☆ | 5 |
| Kodydkova et al. (2009) | ☆☆☆☆ | ☆☆ | 6 |
| Kotan et al. (2011) | ☆☆☆☆ | ☆☆ | 6 |
| Lackovic et al. (2023) | ☆☆☆☆ | ☆☆ | 6 |
| Lindqvist et al. (2014) | ☆☆☆ | ☆☆ | 5 |
| Mahdi et al. (2022) | ☆☆☆☆ | ☆☆ | 6 |
| Oglodek (2017) | ☆☆☆☆ | ☆ | 5 |
| Oglodek et al. (2017) | ☆☆☆☆ | ☆ | 5 |
| Ormonde do Carmo et al. (2015) | ☆☆☆☆ | ☆ | 5 |
| Rybka et al. (2013) | ☆☆☆☆ | ☆☆ | 6 |
| Samaryn et al. (2023) | ☆☆☆☆ | ☆☆ | 6 |
| Sarandol et al. (2007) | ☆☆☆☆ | ☆☆ | 6 |
| Silva et al. (2019) | ☆☆☆ | − | 3 |
| Shungu et al. (2012) | ☆☆☆☆ | ☆☆ | 6 |
| Smith et al. (2021) | ☆☆☆☆ | ☆ | 5 |
| Srivastava et al. (2002) | ☆☆☆☆ | − | 4 |
| Stefanescu et al. (2012) | ☆☆☆☆ | ☆☆ | 6 |
| Tsai et al. (2016) | ☆☆☆☆ | ☆ | 5 |
| Tuura et al. (2023) | ☆☆☆ | ☆ | 4 |

Supplementary Table 2: Results of univariate meta-regression analyses on GSH in whole samples.

| Moderator (number of studies) | Coefficient | 95% CI | p-value |
| --- | --- | --- | --- |
| age (16) | −0.0015 | −0.024 to 0.021 | 0.90 |
| proportion of male (15) | −1.98 | −4.86 to 0.90 | 0.18 |
| proportion of medicated patients (15) | −0.0038 | −0.015 to 0.0073 | 0.50 |
| duration of illness (7) | 0.0065 | −0.0047 to 0.018 | 0.26 |

Abbreviations: CI, confidence interval; GSH, glutathione

Supplementary Table 3: Results of univariate meta-regression analyses on GPx in whole samples.

| Moderator (number of studies) | Coefficient | 95% CI | p-value |
| --- | --- | --- | --- |
| age (18) | −0.010 | −0.034 to 0.0015 | 0.42 |
| proportion of male (17) | 0.75 | −1.31 to 2.82 | 0.47 |
| proportion of medicated patients (16) | −0.0041 | −0.014 to 0.0055 | 0.41 |
| duration of illness (6) | 0.0055 | −0.0084 to 0.019 | 0.44 |

Abbreviations: CI, confidence interval; GPx, glutathione peroxidase

Supplementary Table 4: Results of univariate meta-regression analyses on GR in whole samples.

| Moderator (number of studies) | Coefficient | 95% CI | p-value |
| --- | --- | --- | --- |
| age (8) | −0.0036 | −0.061 to 0.068 | 0.91 |
| proportion of male (7) | 0.70 | −3.26 to 4.65 | 0.73 |

Abbreviations: CI, confidence interval; GR, glutathione reductase
